# Classification of Infection during the Puerperium, with Notes on Treatment

**Published:** 1893-11

**Authors:** R. R. Kime

**Affiliations:** Professor Gynecology Atlanta Polyclinic, Lecturer on Gynecology and Adjunct to Chair of Obstetrics, Southern Medical College, Atlanta, Ga.


### CLASSIFICATION OF INFECTION DURING THE PUERPERIUM, WITH NOTES ON TREATMENT.*

By R. R. KIME, M. D.,

Professor Gynecology Atlanta Polyclinic, Lecturer on Gynecology and Adjunct to Chair of Obstetrics, Southern Medical College, Atlanta.

Much has been written on this subject, many microscopical investigations made, various plans of prophylaxis and treatment advocated, from which great benefit has been derived and many a useful life saved. While this is true there is yet a want of unanimity of opinion and a lack of definiteness in the use of terms in considering the etiology and nomenclature of puerperal infection.

From close observation as to etiology and clinical manifestations it certainly is evident that the terms of puerperal fever and puerperal septicemia are inadequate and should be abandoned as generally used.

It is now generally recognized that other causes than infection may produce fever during the puerperal state and that there is more than one variety of infection; hence the necessity for definite terms to express the different conditions.

Some two years since in an article on this subject I presented the following classification based upon the term puerperal infection as introduced by H. J. Garrigues, of New York, who classes all forms of infection during the puerperal state under the term puerperal infection, which term I think should be divided into two general classes according to cause.

All those cases produced by the absorption of the products of putrefaction should be classed under the head of putrid infection, while those the result of the absorption of septic material and germs strictly, would be classed under septic infection. If shorter terms

*Read before the Atlanta Obstetrical Society September 15.

are desired it would seem advisable to make putrid infection synonymous with sapremia, and septic infection synonymous with septicemia; thus we have:

Puerperal infection { Putrid infection or sapremia.
Septic infection or septicemia.

The question arises, is such a nomenclature sustained by the etiology and clinical manifestations of puerperal infections?

I think so, and that also microscopical demonstrations and treatment of these conditions give additional weight to the advisability of such classification.

Every obstetrician has seen cases in which he has eliminated all possible sources by which septic material might have been conveyed, a few days after labor gradually develop symptoms indicative of infection, which on close investigation give the following history:

First. The pulse rate increasing six to eight or ten beats per day, followed by a gradual elevation of temperature, furred tongue and headache. The uterine cavity will be found to contain material undergoing putrefaction and being absorbed. In such cases clear and disinfect uterine cavity, giving free drainage, and patient will rapidly improve if other complications have not already been produced. As a rule these cases develop slowly, and are less likely to produce pelvic complications and very much more amenable to local treatment. Septic infection gives us as a rule quite a different picture. He who has seen the true cases of septicemia will remember the alarm sounded on the third to the fifth day after labor by a chill, high temperature, elevated pulse, round tongue, often dry, cracked and red on edges, with indications of inflammation in pelvic region; in some distention of abdomen, with indications of peritonitis, in a few cases cessation of lochia. These cases must be met promptly, as they run their course quickly, developing other pelvic complications, in some peritonitis and death. First, clear, cleanse and disinfect uterine cavity, removing all germs and septic material not already absorbed, then give prompt constitutional and supporting treatment, with proper attention to any complications that may arise.

In the first condition, putrid infection, we have the absorption of ptomaines, the result of putrefaction to deal with, its effect depending on the amount absorbed from the original source (uterine cavity) entering circulation, producing effects similar to the action of other alkaloidal poisons. As soon as the source of absorption is removed, the amount taken into system counteracted, the patient is in condition for rapid recovery unless other complications and structural lesions have been produced.

In septic infection (septicemia) we have the introduction and development of septic germs rapidly entering circulation and other pelvic and abdominal structures, producing various complications. The microscope demonstrates the presence of germs in blood and other structures, which is not the case in sapremia, while a fraction of a drop of septic material containing germs will be sufficient to produce devastation and ruin by the rapid development and migration of the germs, as well as by the absorption of leucomaines, their alkaloidal development. In the parturient we have all the environments favorable to the reception and development of infectious material.

To favor sapremia (putrid infection) we have:

1. Often sloughing and putrefaction of tissue, a result of traumatism.

2. The retention and putrefaction of placental tissues and blood clots.

3. Want of drainage, retaining material in uterine cavity that should be thrown off.

4. An open wound at placental site ready for absorption and often raw surfaces resulting from a lacerated cervix, vagina or perineum.

5. A retrograde metamorphosis of tissue to complete involution of generative organs.

The above conditions are often accompanied by a lowered vitality, deranged nervous system and depression due to a prolonged difficult labor, which combined would certainly put patient in a fit condition to be influenced by the absorption of putrid material and much more subject to invasion by septic germs. While the presence of placental tissue, blood-clots and retained secretions are not essential to the development of septicemia as in sapremia, if present they become hotbeds for germ development and should be speedily removed. Without the presence of these the septic germs may ingraft themselves and redevelop from any abraded surface in genital tract. It is thus the accoucheur transmits septic germs by the examining finger or use of instruments.

To place these two varieties of infection in contrast I quote from a former article the following :

| SEPTIC INFECTION, OR SEPTICEMIA | PUTRID INFECTION, OR SAPREMIA. |
|---|---|
| Due to entrance and multiplication of *microbes* and development of *leucomaines*. | Due to absorption of putrefactive *alkaloids* called *ptomaines*. |
| Due to germ life and its reproduction. | Due to absorption of chemical products. |
| | Not contagious. |
| Contagious. | Not inoculable. |
| Inoculable from case to case. | Blood does not contain germs. |
| Blood contains germs similar to those introduced. | |
| May be produced by various germs. | Produced by *ptomaines*, the result of putrefactive bacteria only. |
| May be produced by the fraction of a drop of the virus containing septic germs. | Requires absorption of sufficient chemical products to produce toxic effects similar to toxic doses of poisonous medicines. |
| May be produced by the virus from the exanthematous fevers. | Never. |
| Erysipelas. | Never. |
| Surgical fever. | Never. |
| A previous septic case. | Never. |
| Gonorrhea. | Never. |

There is certainly a marked variation in the clinical history, virulence of the attack and rapid development of complications *produced by* the virus from erysipelas, the exanthemata, a previous septic case as compared with that produced by the absorption of the products of putrid decomposition.

While it is possible for putrid infection, *i. e.*, sapremia, to produce any or all the complications manifested in the pelvic organs as a result of septic infection, it is not the rule, its evolution being slower, with central foci of infection remaining in uterine cavity, producing systemic effects only by absorption of the akaloidal developments, hence the beneficial results from local treatment in this variety of infection.

While we are considering the severer forms of infection, we must not forget there are milder invasions and are more common to sapremia than septicemia, resulting in those conditions so often encountered by the gynecologist, known as chronic endometritis, subinvolution, salpingitis, with their subsequent results, such as adhesions, pelvic peritonitis, pus formation, *i. e.*, pyosalpinx and possibly even the much doubted and misapplied term, pelvic cellulitis.

The expectant plan of treatment *of abortion* is one of the most fruitful sources and commonest causes of putrid infection, as well as failing to thoroughly empty uterus after labor.

We have only partially presented the etiological and clinical evidences in favor of distinguishing between putrid and septic infection, to which many points of confirmation might be added from microscopical demonstrations and chemical analysis. While we contend for a distinction between septicemia and sapremia, we also recognize the possibility of a mixed infection and the difficulty in a differential diagnosis, yet this is true of other distinct and separate diseases.

The generative organs are the gateway by which infection enters, and if properly quarantined infection will not occur. No case is autogenetic, not even those of putrid infection, for the blood-clot, retained membranes or secretions must decompose before they become infectious, and bacterial invasion is essential to putrefaction. In cases due to ruptured pus tubes or gonorrhea the infectious material was previously introduced, possibly before intervention of pregnancy.

The primary foci of infection is always in the generative organs, and usually the uterus; hence the necessity to invariably investigate the condition of these parts, removing any infectious materal that may be found in vaginal canal or uterus. This procedure is essential in all cases of *septic* as well as *putrid* infection. If dilatation of cervix with irrigation removes all infectious material, then disinfect parts.

If irrigation fails to remove the offending material, then it should be removed by curette, forceps and curette, using *antiseptic irrigations before and after removal.* Before, to wash out excess and disinfect remaining portions of infectious material as far as possible before using curette, making new abrasions and opening new channels for absorption. After, to more completely remove infectious material and disinfect uterine cavity.

These irrigations should be made with hot solutions of mercuric chloride 1 in 3 to 5,000, carbolic acid 5 per cent. solution, saturated boric acid solution, a 2 to 5 per cent. creoline solution or a weak iodine solution.

Always follow the mercuric chloride irrigation with hot water, to prevent absorption. After this procedure the most efficient application to endometrium in my hands has been the camphorated phenique, *i. e.*, equal parts of carbolic acid and gum camphor, well-rubbed together, one-half to one ounce thrown up into the uterus by a long flexible metal nozzle, attached to hard rubber syringe, an ordinary male glass syringe with lumen catheter, new, or at least clean, may be substituted. An intrauterine suppository of iodoform and boric acid will aid materially in disinfecting. If preferred tincture iodine may be used in same manner. To prolong the disinfection of uterus, a suppository containing iodoform and boric acid may be introduced into uterine cavity. An excellent suppository would be one containing camphorated phenique gtts. x, iodoform gr. x, boric acid gr. x, cocoa butter q. s. to make one suppository.

Iodoform gauze packing is advised by some; while it acts admirably in other conditions with small uterus and only serum to be drained off, it has obstacles to overcome in these cases. We have a large uterus, increased excretions, degeneration of endometrium, broken down tissues and cells to be removed, which the gauze cannot do, besides the drainage action may be reversed where gauze is loosely packed in uterus for several hours and not subject to pressure with accumulation of fluid in vagina. Thus the current may be from vagina to uterus, the gauze not only obstructing the throwing off of material from uterine cavity, but actually carrying fluid into it. A strip of gauze, extending from uterine cavity to vulva, with a septic absorbent vulval pad might obviate this difficulty to some extent.

The irrigations, with dilatation of cervix and applications, should be repeated from two to three or four times in twenty-four hours, sufficiently often to secure *thorough drainage* and disinfection of uterine cavity.

The beneficial results of this local treatment are most marked in putrid infection; while they are useful and essential in septic infection, they will only destroy or remove those septic germs found in uterine cavity or endometrium, and most essential at the beginning of septicemia. In this variety of infection, when there is but little discharge from uterus, pulse and temperature not lowered by their use, they should be used *less frequently or discontinued entirely.*

Constitutional treatment in septicemia should be actively and energetically carried out, while it is less essential in sapremia, the local treatment being more important.

At first the following may be used to advantage:

℞. Calomel trit. (1 in 4), gr. x.
Phenacetine, gr. x.
Quinine, ʒjss.

M. Divide into x powders.

Sig. One every four hours. Combined with moderate use of salines.

Opiates should be avoided in these cases if possible. Ergot is disappointing by contracting circular fibers of internal os, preventing drainage. Ergot has but little use in obstetric practice.

For continued use a combination of nux vomica, tr. cinchona comp., ess. pepsin and brandy every four hours has done me good service.

To follow use of quinine some form of iron would be beneficial. I often prescribe, where stomach will retain it,

℞. Tr. chloride of iron, ʒ ij.

Hydrochloric acid, c. p., ʒ ss.

Mercuric chloride, gr. j.

Ess. pepsin, ℥ ij.

Glycerine, q. s. ℥ jv.

M. Sig. One teaspoonful in wineglassful water every four hours during the day.

In some cases turpentine, in form of an emulsion, acts well, best administered during the night.

All cases should be fed systematically from the beginning liquid, easily digested food. If they cannot retain and assimilate it, some of the prepared foods should be substituted, such as beef peptonoids, wine and iron, Wyeth's Beef Juice, peptonized or malted milk.

Antipyretics, especially of the coal tar group, should never be used as antipyretics except in cases of extreme necessity, for they, like opium, do not eradicate or counteract the poison, but obscure the symptoms and lull the physician and patient into a false hope of security. Remove the *materies morbi*, and, as a rule, the temperature, pain, etc., will disappear, but do not kill patient by depressing antipyretics or checking elimination by opiates. The coal tar derivatives are beneficial in these cases as analgesics, but obscure symptoms.

In the use of the sharp curette it should be limited to those cases in which the *uterine walls are not* degenerated and softened or sloughing for fear of perforating wall of uterus.

If cervix is properly dilated, uterus properly irrigated and disinfected *before* and *after* use of curette, there need be but little fear of increased absorption in any case, especially if uterus is *kept properly drained and disinfected.*

Abdominal section in these cases is a more difficult question to decide. When to operate and when not to operate is a cardinal question.

By prompt operative measures lives may be saved; by delay lives will be sacrificed in some instances, while in others the delay will prevent the erection of a monument over the remains of the surgeon's work. Happy is the physician who makes no mistake in his diagnosis, and promptly recognizes the indications for operative measures. This is a fertile field for future development. At present operative measures are advised principally on the basis of individual opinions and experience.

With a view of eliciting some discussion on this subject, I present the following propositions.

As a rule, never perform celiotomy until uterus has been thoroughly emptied, disinfected and drained, adding free use of copious hot water vaginal douches and establishment of the eliminative exosmotic and cathartic action of salines with proper constitutional treatment. When the above has been conscientiously carried out without relief to patient, then the following operative measures should be advised unless there are conditions and pathological lesions present contraindicating such procedures:

1st. Operate in all cases, flushing abdomen and giving free drainage, as soon as general peritonitis is diagnosed, if vitality of patient is sufficient to endure the shock.

2d. Operate in all cases where there is *severe constitutional symptoms with indications of shock or rapid absorption of septic material,* where the condition of pelvic organs is not sufficient to account for the marked constitutional disturbance, but sufficient evidence to establish diagnosis of general peritonitis.

3d. Operate in all cases where pyosalpinx or ovarian abscess is produced, provided there is not also present *extensive* lymphangitis or phlebitis.

4th. Hysterectomy may be considered where there is degeneration, sloughing or pus formation in the uterine muscular structures, with or without involvement of tubes and ovaries.

5. Operate in all cases where a pyosalpinx, ovarian abscess or any cystic tumor has ruptured into abdominal cavity as the result of labor.

Celiotomy is certainly too grave a procedure to be entered into lightly or recklessly in these cases. The physician, gynecologist or surgeon who would advise abdominal section in puerperal infection because he did not know what else to do, is not only unworthy the confidence of his patient, but is a daring, reckless, unconscientious experimenter.

*Celiotomy should never be performed in these cases unless the local pelvic condition demands it or the constitutional condition is such as to indicate a general peritonitis or rapid absorption of infectious material from abdominal cavity.*

Such expressions as the following and from so eminent an authority as A. Lapthorn Smith, M. D., Montreal, Canada (*New*

*York Journal Gynecology and Obstetrics*, November, 1892, page 1026): "Whether we are able to feel anything or not, if the temperature has not come down within twenty-four hours after curetting, our next step must be an exploratory incision. * * * * * * * In another case (A. L. Smith, M. D.) the writer opened the abdomen and found nothing to account for the disease, and he accordingly removed the infected uterus, which was saturated with pus, and the patient made a good recovery"—certainly are too broad and radical, and if universally heeded will result in many useless operations and the death of as many puerperal women as it will save.

No physician, be he a master surgeon or not, is justifiable in performing celiotomy and removal of the uterus where "he cannot find anything to account for the disease," although in *one case the condition of the uterus justified the operation.* How would it be with a score of others, especially in the hands of less expert operators? Every surgeon and gynecologist should be ready and willing to assume the responsibilities of operative measures in these cases when the pathological lesions, physical condition and constitutional symptoms demand heroic measures, thereby saving life and inspiring confidence in surgical work by the general public as well as the profession.

To do this we must have definite ideas as to indications and contraindications and what may be accomplished by operative measures.

To remove pus tubes and leave behind an infected uterus, with muscular walls degenerated and sloughing or infiltrated with purulent material, would not benefit patient nor be justifiable; nothing but a hysterectomy would meet the indications.

It is certainly plausible and possible that infection during the puerperium may produce a lymphangitis, with inflammation of the adjacent cellular tissue, which may break down, forming pus in the broad ligament. If such be the case (which is denied by some), then the life of patient would be most likely preserved by vaginal drainage, and later, when vitality of patient is better and pelvic condition improved, perform celiotomy if the condition of the patient demand it.

Many of these cases nature drains by rupture into vagina, or occasionally by rupture in inguinal region near Poupart's ligament. Celiotomy certainly is not advisable during the inflammatory or formative stage for all the infected tissue and lymphatics could not be removed and by drainage through the vagina the peritoneal cavity is not opened or infected, and if the case is not relieved, later celiotomy can be performed with more prospect of removing the diseased tissues and saving the life of the patient.

Abdominal section should not be advised until there are positive indications demanding it.

To illustrate, a woman was attended in confinement by one of the leading physicians in this city, followed by two others, when the fourth, a gynecologist, was consulted, who advised abdominal section. The case had elevation of pulse 120 to 140, temperature, 102° to 104$\frac{1}{5}$° F., bowels distended with gas, tongue small, round and red on edges, with some thickening, and slight exudate in broad ligaments, yet no localized accumulation of fluid could be mapped out.

Under tonics, antimalarials and restricted diet symptoms disappeared in eight or ten days to be followed by a relapse in fourteen days, with pulse 120, temperature 104. By free use of antimalarials temperature and pulse dropped to normal in forty-eight hours. If abdomen had been opened, "nothing found to account for the disease," hysterectomy performed, another success for pelvic surgery would have been "scored."

In conclusion I would advise in all cases of puerperal infection, first, a thorough cleansing and disinfection of uterus, keeping it drained and disinfected with proper constitutional supportive treatment, and then if the symptoms, pathological lesions and physical condition of the patient demand abdominal section, do it promptly, as the several indications may demand, with proper attention to the details of the operative technique, eliminating as far as possible all errors in diagnosis, entertaining a due regard for contraindications to such operative measures.

